# The impact of bariatric surgery on male sexual function: a systematic review and meta-analysis

**DOI:** 10.3389/fendo.2026.1798050

**Published:** 2026-03-25

**Authors:** Zhongjian Qin, Weizhen Wu, Yongqiang Wei, Hanyu Xu, Baoxing Liu, Binghao Bao

**Affiliations:** 1 Graduate School of Beijing University of Chinese Medicine, Beijing, China; 2 Department of andrology, China-Japan Friendship Hospital, Beijing, China

**Keywords:** bariatric surgery, erectile dysfunction, male sexual function, meta-analysis, obesity

## Abstract

**Background:**

Obesity has become a major global public health challenge, closely associated not only with metabolic and cardiovascular disorders but also with male sexual dysfunction. Emerging evidence suggests that bariatric surgery may improve sexual function in obese men by enhancing hormonal balance, reducing visceral adiposity, and restoring endothelial function. However, existing studies show strong heterogeneity in design, surgical approaches, and sample sizes, underscoring the need for an updated systematic review and meta-analysis.

**Objective:**

To systematically evaluate and meta-analyze the effects of bariatric surgery on sexual function and related hormonal levels in obese men.

**Methods:**

PubMed, Web of Science, Embase, and the Cochrane Library were systematically searched for relevant studies published up to June 2025. A random-effects model was applied to pool effect sizes, and heterogeneity was assessed using the I² statistic. Changes in International Index of Erectile Function (IIEF) scores and hormone levels before and after surgery were evaluated. Subgroup analyses, sensitivity analyses, and publication bias assessments were conducted, with all analyses performed using Stata 17.0.

**Results:**

A total of 21 studies involving 695 participants were included. Pooled analyses showed significant improvements in both IIEF-5 and IIEF-15 total scores after surgery (MD = 6.45 and 9.03, respectively; p < 0.001), indicating overall enhancement in sexual function. Domain-specific results demonstrated significant improvements in erectile function, orgasmic function, sexual desire, intercourse satisfaction, and overall satisfaction, with erectile function showing the greatest improvement (SMD = 0.76); however, differences between domains were not statistically significant. Regarding hormonal outcomes, total testosterone increased significantly after surgery (MD = 8.21, p < 0.001), whereas free testosterone showed no significant change. Body mass index (BMI) decreased markedly (MD = -13.86, p < 0.001). Subgroup analyses consistently supported the beneficial effects of surgery. Sensitivity analyses confirmed the robustness of the results, and Egger’s test indicated no significant publication bias.

**Conclusions:**

Bariatric surgery significantly improves sexual function in obese men, particularly erectile function, and increases total testosterone levels. These findings suggest that, beyond weight reduction and metabolic improvement, bariatric surgery may also play an important role in enhancing sexual health and overall quality of life. Further studies, including randomized controlled trials with standardized hormonal assessment and extended follow-up, are required to establish the causal relationship between surgery and sexual function.

## Introduction

Obesity has become a global public health problem, with its prevalence steadily increasing over the past decades, imposing substantial burdens on metabolic, cardiovascular, and psychological health (1, 2). A growing body of evidence demonstrates that obesity is not only closely associated with diabetes, hypertension, and dyslipidemia, but also exerts adverse effects on male sexual health (3). Epidemiological studies have shown that obese men are at significantly higher risk of erectile dysfunction (ED) compared with men of normal weight (4). The underlying mechanisms involve multiple pathways, including endothelial dysfunction, chronic low-grade inflammation, decreased androgen levels, and psychological factors (5–7).

Weight reduction is a core strategy for improving obesity-related comorbidities. While lifestyle modification and pharmacotherapy play important roles in weight management, their long-term efficacy is limited in patients with severe obesity (8). In recent years, bariatric surgery has emerged as an important therapeutic option for severe obesity and metabolic syndrome, given its remarkable benefits in weight control, metabolic improvement, and cardiovascular risk reduction. Common procedures include Roux-en-Y gastric bypass (RYGB), sleeve gastrectomy (SG), adjustable gastric banding (AGB), and biliopancreatic diversion (BPD).

Previous studies have suggested that bariatric surgery may improve male sexual function and quality of life by enhancing androgen levels, reducing visceral adiposity, and restoring endothelial function (9, 10). Several systematic reviews and meta-analyses have reported significant improvements in the International Index of Erectile Function (IIEF) and its subdomain scores following bariatric surgery (11, 12). However, the available evidence remains limited: differences exist in study design; the effects of various surgical procedures on sexual function may differ; and some studies included relatively small sample sizes.

Therefore, it is necessary to integrate the latest evidence to provide a more comprehensive understanding of the impact of bariatric surgery on male sexual function. The present study aimed to systematically review and quantitatively evaluate the effects of bariatric surgery on male sexual function, with the IIEF-5 and IIEF-15 as primary outcomes and testosterone levels and body mass index (BMI) as secondary outcomes, in order to provide higher-quality evidence for clinical practice.

## Methods

### Search strategy

This study was conducted in accordance with the Preferred Reporting Items for Systematic Reviews and Meta-Analyses (PRISMA) guidelines (13). A systematic search was performed in four databases: PubMed, Web of Science, Embase, and the Cochrane Library, covering publications up to June 2025. The search terms included two categories: (i) interventions: bariatric surgery, sleeve gastrectomy, gastric bypass, gastric banding, biliopancreatic diversion; and (ii) outcomes: sexual function, erectile dysfunction. Grey literature was also searched to minimize the risk of missing relevant studies. The review was registered with PROSPERO (CRD420251062697).

### Inclusion and exclusion criteria

Inclusion criteria:

1. Adult male participants (≥18 years) undergoing bariatric surgery;
2. Interventions included sleeve gastrectomy, gastric bypass, gastric banding, or biliopancreatic diversion;
3. Eligible study designs included randomized controlled trials (RCTs), prospective or retrospective cohort studies, before-and-after self-controlled studies, and cross-sectional comparative studies;
4. Primary outcomes included the International Index of Erectile Function (IIEF-5 or IIEF-15);
5. Studies provided extractable data (mean, standard deviation, and sample size, or other convertible effect size data).

Exclusion criteria:

1. Studies that did not distinguish male participants;
2. Studies without bariatric surgery as the intervention (pharmacotherapy, lifestyle modification);
3. Studies without control or pre–post comparison;
4. Studies that did not report sexual function outcomes or did not provide extractable effect size data; case reports, reviews, meta-analyses, conference abstracts, or editorials;
5. Duplicate publications or overlapping data (in such cases, the study with the largest sample size or most complete data was included).

### Data extraction

Two investigators independently and blindly extracted data using a predefined form. Discrepancies were resolved by discussion with a third investigator. Extracted information included: first author, year of publication, country/region, study design, sample size, age, body mass index (BMI), type of bariatric surgery (SG, RYGB, AGB, BPD), follow-up duration, and mean and standard deviation of IIEF-5and IIEF-15 before and after surgery. Data on total testosterone (TT) and free testosterone (FT) before and after surgery were also collected.

If a study included multiple surgical procedures or multiple outcomes, relevant data were extracted separately. All data were cross-checked by two investigators to ensure accuracy.

### Quality assessment

Quality assessment tools were selected according to study design. Cohort, case-control, and cross-sectional studies were evaluated using the Newcastle–Ottawa Scale (NOS), which includes three domains: selection (4 points), comparability (2 points), and outcome assessment (3 points), with a total score of 9. A score ≥7 indicated high quality, 5–6 moderate quality, and <5 low quality (14). Self-controlled before–after studies were assessed using the NIH Quality Assessment Tool for Before–After (Pre–Post) Studies (NIH BA Tool), which consists of 12 items covering study objectives, inclusion criteria, sample size, outcome measurement, follow-up, and reporting completeness. Studies were categorized as high quality (≥70% “yes”), moderate quality (50–69%), or low quality (<50%) (15).

All studies were independently assessed by two investigators, with disagreements resolved through discussion with a third reviewer.

### Statistical analysis

All statistical analyses were conducted using Stata version 17.0. For continuous outcomes, mean difference (MD) with 95% confidence intervals (95% CI) was used as the effect size. When only medians and interquartile ranges were reported, statistical methods were applied to estimate means and standard deviations (16). For before–after studies, pre- and postoperative means and standard deviations were used to calculate the standard error (SE) of change scores, assuming a correlation coefficient of r = 0.5 between pre- and post-surgery measures (sensitivity analyses used r = 0.3 and r = 0.7). For cohort and cross-sectional comparative studies (two-arm studies), MD and SE were calculated based on the difference between intervention and control groups (or postoperative vs. control groups). All effect sizes were converted to MD and SE before pooling.

The main analysis used a random-effects model (DerSimonian–Laird method) to account for potential between-study differences. When heterogeneity was low (I² < 50%), results from the fixed-effect model were also reported. Heterogeneity was assessed using Cochran’s Q test and the I² statistic, with p < 0.10 or I² > 50% indicating significant heterogeneity.

Additional analyses included: Subgroup analysis: stratified by study type (before–after vs. cohort studies) and, where data permitted, by surgical procedure (SG, RYGB, AGB, BPD).Sensitivity analysis: performed by sequentially removing individual studies to evaluate the robustness of pooled estimates and heterogeneity, and by varying the assumed correlation coefficient (r = 0.3, 0.7) in paired studies. Publication bias assessment: conducted when ≥10 studies were included, using Egger’s regression test. All statistical tests were two-tailed, with significance set at p < 0.05.

Given that a significant portion of the included studies adopted a single-arm pre-post design, we will use the same scale design for comparison to minimize errors. We acknowledge that such designs may have regression to the mean effect, placebo effect, and time-related confounding factors, which could lead to an overestimation of the treatment effect.

## Results

### Study selection results

**Figure 1:**
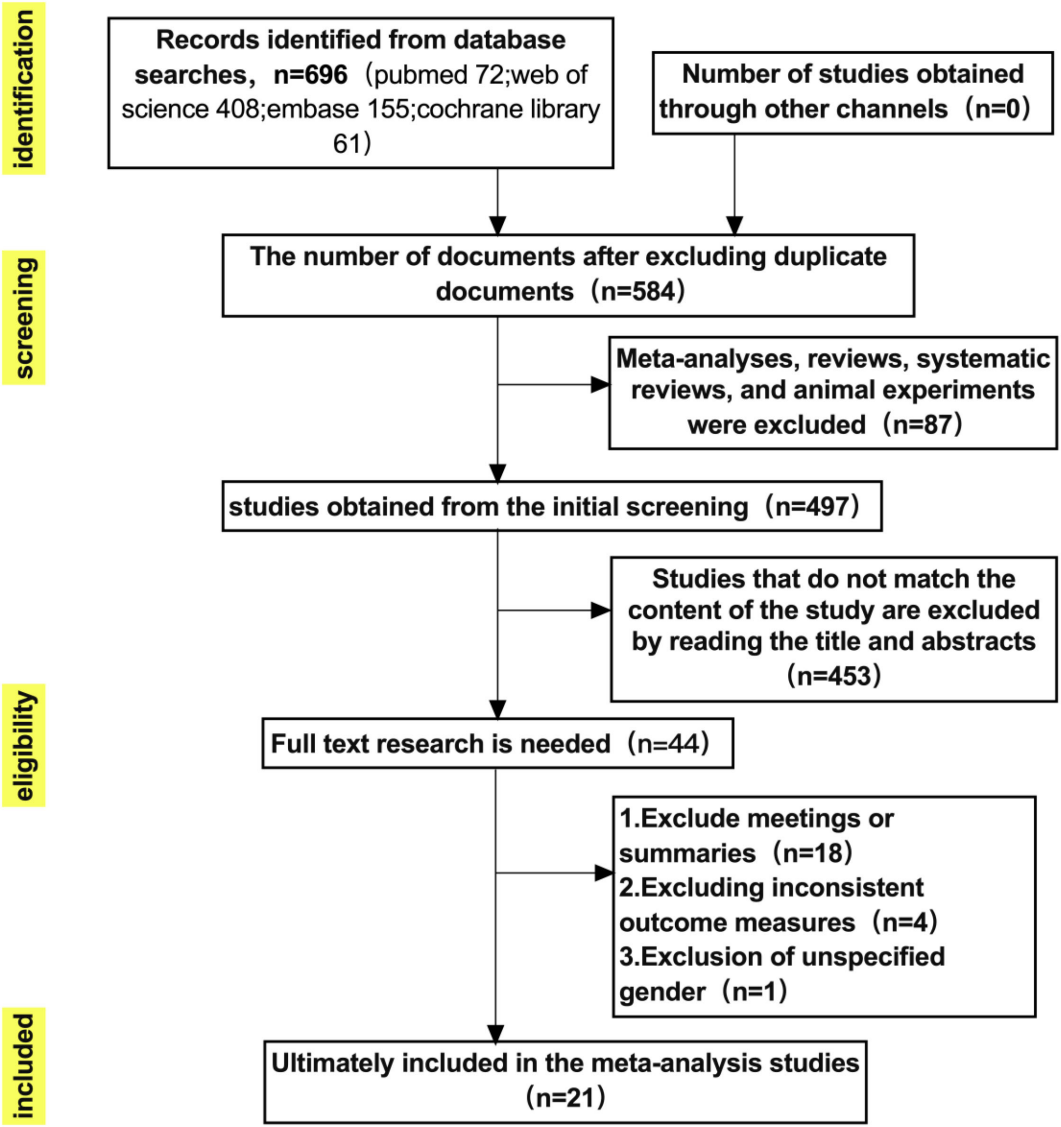
Literature search and selection process. Subsequently, 44 full-text articles were assessed for eligibility. Of these, 18 were excluded because they were conference abstracts or lacked complete data, 4 were excluded due to inconsistent outcome measures, and 1 was excluded for not clearly reporting participants’ sex. Finally, 21 studies met all predefined inclusion criteria and were included in the quantitative synthesis (meta-analysis).

The study selection process is summarized in **Figure 1**. A total of 696 records were initially retrieved through systematic searches of four electronic databases (PubMed, Web of Science, Embase, and the Cochrane Library). After removing 112 duplicates, 584 studies remained for initial screening. Based on titles and abstracts, 87 studies were excluded because they were meta-analyses, reviews, systematic reviews, or animal experiments. The remaining 497 records were further screened, and 453 were excluded as irrelevant to the research topic.

### Baseline characteristics of included studies

This systematic review and meta-analysis included a total of 21 studies published between 2010 and 2025, comprising prospective cohort studies (PC), retrospective cohort studies (RC), cross-sectional studies (CS), and one randomized controlled trial (RCT). The studies were conducted across multiple countries and regions, including the United States, China, Brazil, Australia, Turkey, Poland, Egypt, Iran, Spain, the United Kingdom, Greece, and Malaysia. The participants ranged in age from 22 to 64 years, with a total of 695 men undergoing bariatric surgery.

The surgical procedures involved included sleeve gastrectomy (SG), Roux-en-Y gastric bypass (RYGB), adjustable gastric banding (AGB), and biliopancreatic diversion (BPD). Several studies included more than one surgical type. The follow-up period ranged from 6 months to 4 years, with a median and most frequently reported duration of 12 months. For studies that reported multiple follow-up points, we selected the time point closest to 12 months for analysis.

Most studies reported patients’ baseline body mass index (BMI), which ranged approximately from 33.0 to 59.8 kg/m². Regarding study design, about half of the included studies adopted a before–after design, while some also conducted between-group comparisons. Overall methodological quality was relatively high: according to the appropriate quality assessment tools used for each study design, 16 studies were rated as “high quality” and 5 as “moderate quality,” with no study classified as low quality.

Taken together, the included studies encompassed diverse populations and surgical approaches, providing a comprehensive evidence base for evaluating the impact of bariatric surgery on male sexual function (IIEF-5, IIEF-15, and related measures) (**Table 1**).

**Table 1:** Basic characteristics of various studies.

First author	Publication year	Study type	Comparison	Country	Age	Sample size	Surgical procedure	Follow-up time	BMI	Outcome	Quality score	Quality rating
L. O. Reis (17)	2010	RCT	Before vs after and between groups	Brazil	36.7 ± 11.5	10	RYGB	4, 24 months	55.7 ± 7.8	AHI	7/9	High
W. K. B. Ranasinghe (18)	2011	RC	between groups	Australia	52.8 ± 9.33	34	AGB	32 months	47.3 ± 12.67	B	8/9	High
A. Rosenblatt (19)	2013	PC	Before vs after and between groups	Brazil	30-60	23	RYGB	12 months	59.8 ± 12.1	BHI	7/9	High
​M. Mora (20)	​2013​	PC	Before vs after	​Spain​	​43.5 ± 10.3	39	SG/RYGB	​12 months​	46.90 ± 7.77	BCDEFGHI	10/12	High
D. B. Sarwer (9)	2014	PC	Before vs after	USA	24-64	32	RYGB	1, 2, 3, 4 years	45.1 ± 7.56	BCDEFGHI	9/12	High
L. Kun (21)	2015	PC	Before vs after	Poland	45.2	39	RYGB	12 months	46.4 ± 7.8	A	9/12	High
​V. Efthymiou (22)	​2015​	PC	Before vs after	​Greece​	​37.3 ± 9.6​	​30​	SG/BPD/RYGB	​12 months​	_	BCDEFG	8/12	Medium
​M. R. Janik​ (23)	​2016​	CS	between groups	​Poland​	​43 ± 10​	​30​	SG/RYGB	​12 months​	_	BCDEFG	7/9	High
​M. Aleid​ (24)	​2017​	PC	Before vs after	​UK​	​48.9 ± 7.0​	18	AGB/SG/RYGB	​1, 3, 6 months​	​46.8 ± 17.78​	BCDEFG	10/12	High
H. F. Oncel (25)	2020	PC	Before vs after	Turkey	28-49	40	SG	6 months	47.2 ± 6.62	AH	9/12	High
F. C. Karaca (26)	2020	PC	Before vs after	Turkey	50.5 ± 6.6	36	SG	6 months	33.01 ± 3.06	BCDEFG	10/12	High
Fahmy (10)	2021	PC	Before vs after	Egypt	34.7 ± 8.6	65	SG	12 months	40.2 ± 3.8	BCDEFG	8/12	Medium
F. Gokalp (27)	2021	PC	Before vs after	Turkey	34 (28,38)	31	SG	12 months	50.1 ± 4.08	BCDEFGH	8/12	Medium
M. D. Sarhan (28)	2021	PC	Before vs after	Egypt	22-60	48	SG/RYGB	12 months	52.03 ± 9.49	BCDEFGH	11/12	High
​F. P. Machado​ (29)	​2021​	PC	Before vs after	​Brazil​	​36.3 ± 8.1​	​33​	SG/RYGB	​6 months​	​43.8 ± 7.8​	BCDEFGHI	10/12	High
G. Chen (30)	2022	PC	Before vs after and between groups	China	30.8 ± 7.3/32.8 ± 6.9	18/19	SG/RYGB	12 months	41.98 ± 7.89	BCDEFGH	7/9	High
​I. M. Ambres​ (31)	​2022​	PC	Before vs after	​Spain​	​45 ± 4.87​	​12​	SG/RYGB	​6, 12, 18 months​	​42.37 ± 4.44​	BCDEFGHI	9/12	High
Mohamed Hamed.Sultan (32)	2023	PC	Before vs after and between groups	Malaysia	30-45	13	Unknown	1, 3, 6 months	44.1 (41-56)	A	7/9	High
F. Nosrati (33)	2023	PC	Before vs after	Iran	39.4 ± 9.2	41	SG/RYGB	12 months	47.15 ± 5.4	BCDEFG	8/12	Medium
X. Gao (34)	2024	PC	Before vs after	China	25.59 ± 3.7	34	SG	3, 6, 12 months	37.42 ± 3.64	AH	10/12	High
P. Malczak (35)	2025	PC	Before vs after	Poland	43.9 ± 8.4	60	SG	12 months	48.8± 7.1	CHI	6/9	Medium

A, IIEF-5; B, IIEF-Total; C, IIEF-Erectile Function; D, IIEF-Sexual Desire; E, IIEF-Orgasm Function; F, IIEF-Intercourse Satisfaction; G, IIEF-Total Satisfaction; H, Total testosterone; I, Free testosterone; PC, prospective cohort; RC, retrospective cohort RCT, randomized clinical trial; CS, Cross-sectional study; RYGB, Roux-en-Y gastric bypass; SG, sleeve gastrectomy; BPD, biliary pancreatic diversion; AGB, Adjustable gastric band.

According to the NOS, seven two-arm studies were evaluated for methodological quality (**Table 2**). The results indicated an overall good quality. Six studies were rated as high quality, while two studies were rated as moderate quality.

**Table 2:** Evaluation of literature quality (NOS).

First author	Year	NOS score			Total score	Quality rating
		Selection	Comparability	Outcome/composure		
Sultan (32)	2023	4★	1★	2★	7	High
Rosenblatt (19)	2013	4★	1★	2★	7	High
Janik (23)	2016	4★	2★	1★	7	High
Reis (17)	2012	4★	1★	2★	7	High
Ranasinghe (18)	2014	4★	2★	2★	8	High
Chen (30)	2019	4★	1★	2★	7	High
Malczak (35)	2020	4★	0★	2★	6	Medium

The symbol "★" represents a score of one point in the Newcastle-Ottawa Scale (NOS).

In addition, the NIH BA Tool was applied to assess the quality of the included single-arm studies (**Table 3**). The evaluation showed that 10 studies were rated as high quality, whereas 4 studies were rated as moderate quality.

**Table 3:** Evaluation of literature quality (NIH BA Tool).

First Author	Year	Score per Item (Q1–Q12)	Total Score (0–12)	Quality Rating
L. Kun (21)	2014	[1, 1, 1, 1, 1, 0, 1, 1, 0, 1, 1, 0]	9	High
M. D. Sarhan (28)	2021	[1, 1, 1, 1, 1, 1, 1, 1, 1, 1, 1, 0]	11	High
F. Nosrati (33)	2023	[1, 1, 1, 1, 1, 0, 1, 0, 0, 1, 1, 0]	8	Medium
M. Mora (20)	2013	[1, 1, 1, 1, 1, 1, 1, 1, 0, 1, 1, 0]	10	High
I. Miñambres (31)	2022	[1, 1, 1, 1, 1, 0, 1, 1, 0, 1, 1, 0]	9	High
Halil F. Öncel (25)	2015	[1, 1, 1, 1, 1, 0, 1, 1, 0, 1, 1, 0]	9	High
Fahmy (10)	2016	[1, 1, 1, 1, 1, 0, 1, 0, 0, 1, 1, 0]	8	Medium
X. Gao (34)	2017	[1, 1, 1, 1, 1, 1, 1, 1, 0, 1, 1, 0]	10	High
F. Gokalp (27)	2018	[1, 1, 1, 1, 1, 0, 1, 0, 0, 1, 1, 0]	8	Medium
F. C. Karaca (26)	2020	[1, 1, 1, 1, 1, 1, 1, 1, 0, 1, 1, 0]	10	High
M. Aleid (24)	2019	[1, 1, 1, 1, 1, 1, 1, 1, 0, 1, 1, 0]	10	High
V. Efthymiou (22)	2020	[1, 1, 1, 1, 1, 0, 1, 0, 0, 1, 1, 0]	8	Medium
F. P. Machado (29)	2021	[1, 1, 1, 1, 1, 1, 1, 1, 0, 1, 1, 0]	10	High
D. B. Sarwer	2008	[1,1,1,1,1,0,1,1,0,1,1,0]	9	High

### Meta-analysis results

#### Primary outcomes

##### IIEF-5

**Figure 2:**
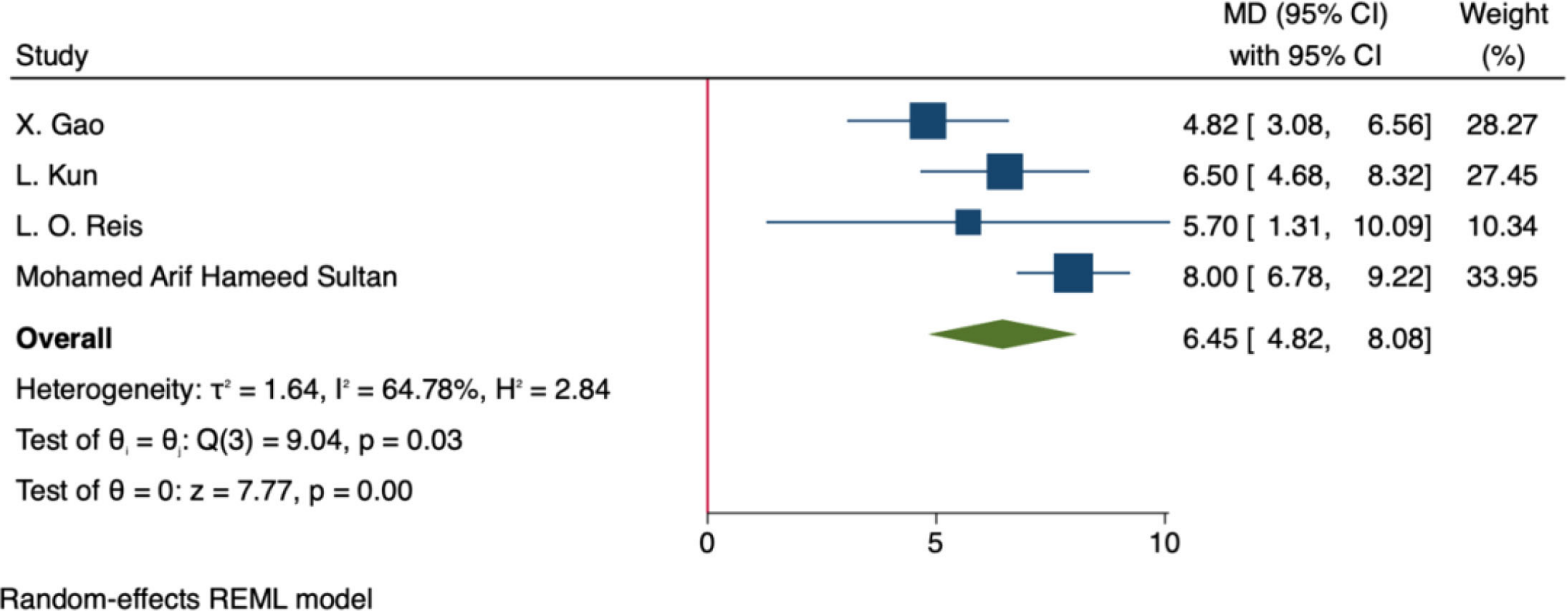
Forest plot-comparison of preoperative and postoperative International Index of Erectile Function-5.

A total of four studies used the IIEF-5 score as an indicator of male sexual function. The pooled analysis based on a random-effects model demonstrated that bariatric surgery significantly improved IIEF-5 total scores, with a combined effect size of MD = 6.45, 95% CI: 4.82–8.08, p < 0.001. Heterogeneity analysis indicated moderate heterogeneity among studies (I² = 64.8%, p = 0.03) (**Figure 2**).

##### IIEF-15

**Figure 3:**
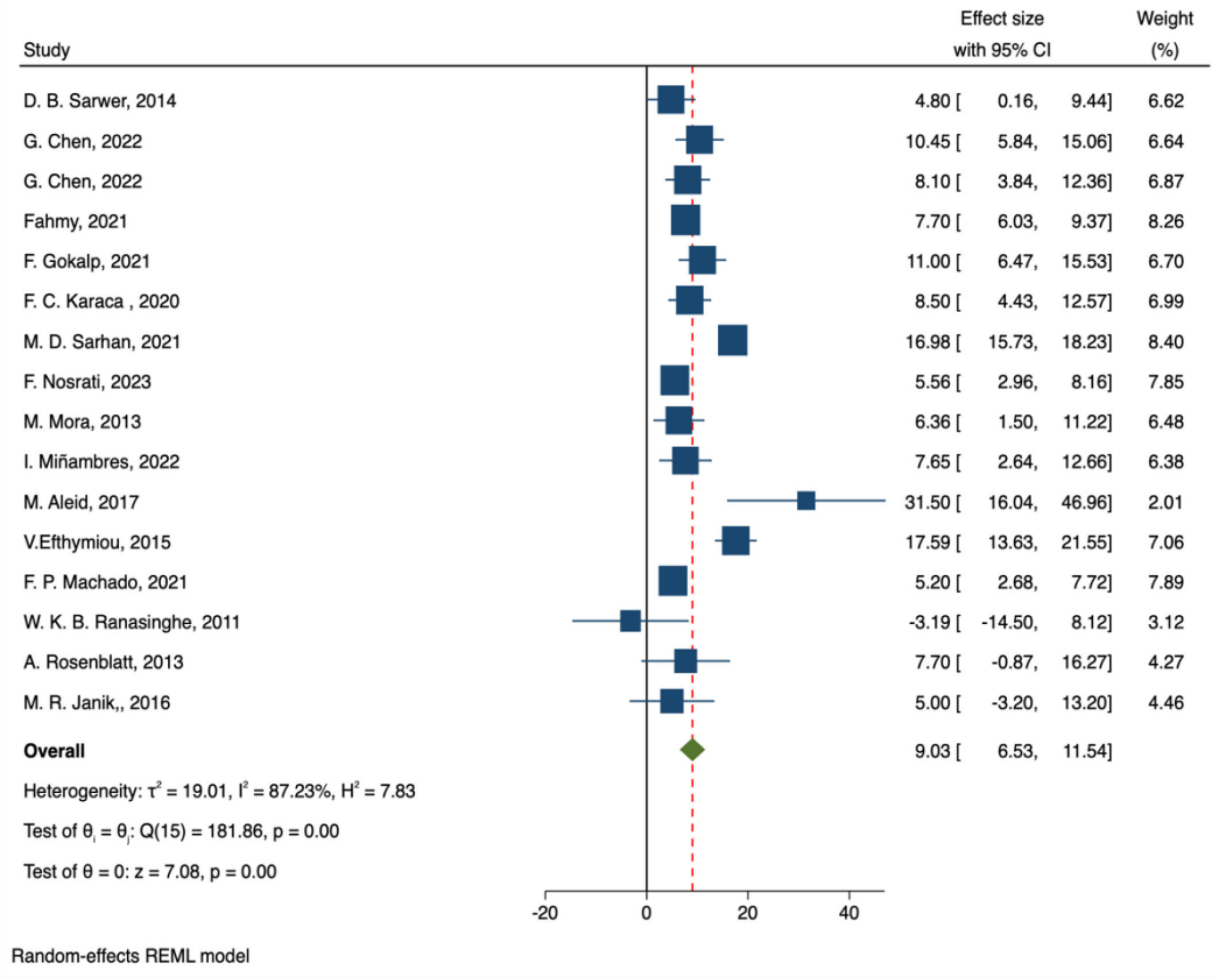
Forest plot-comparison of preoperative and postoperative International Index of Erectile Function-15.

A total of 16 studies assessed the IIEF-15 as an outcome measure. Ranasinghe et al. (18) reported that bariatric surgery decreased IIEF-15 total scores, whereas the remaining studies indicated an increase. The pooled results from a random-effects model demonstrated that bariatric surgery significantly improved IIEF-15 total scores, with a combined effect size of MD = 9.03, 95% CI: 6.53–11.54, p < 0.001. Heterogeneity analysis revealed substantial heterogeneity across studies (I² = 87.2%, p < 0.001) (**Figure 3**).

##### IIEF- erectile function

**Figure 4:**
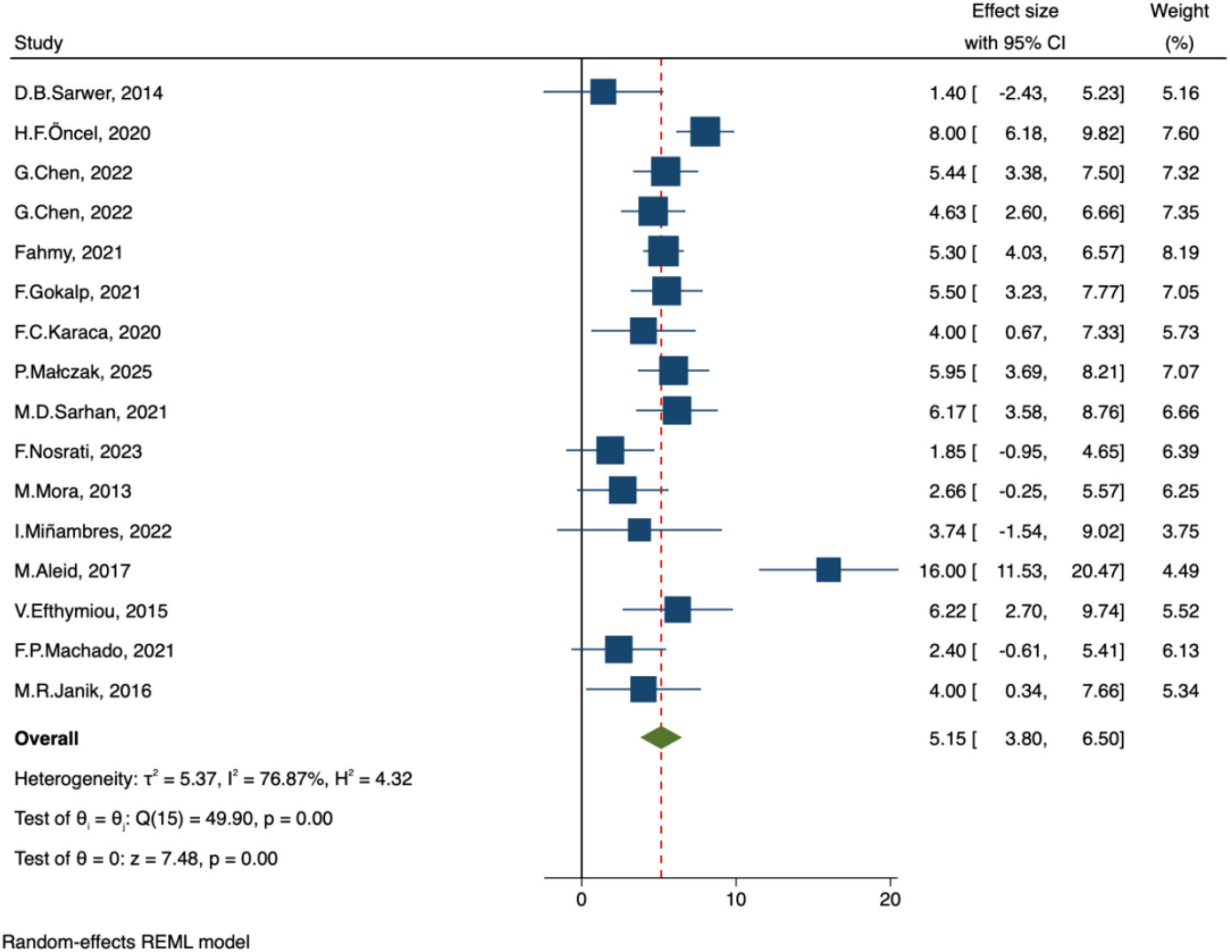
Forest plot-comparison of preoperative and postoperative International Index of Erectile Function-Erectile Function.

In our included studies, a total of 16 studies evaluated the effect of bariatric surgery on male erectile function, as measured by the IIEF-EF domain. The pooled results from a random-effects model indicated that bariatric surgery significantly improved erectile function, with a combined effect size of MD = 5.15, 95% CI: 3.80–6.50, p < 0.001. Heterogeneity testing suggested considerable variability among studies (I² = 76.9%, p < 0.001) (**Figure 4**).

##### IIEF-orgasm function and IIEF- desire

**Figure 5:**
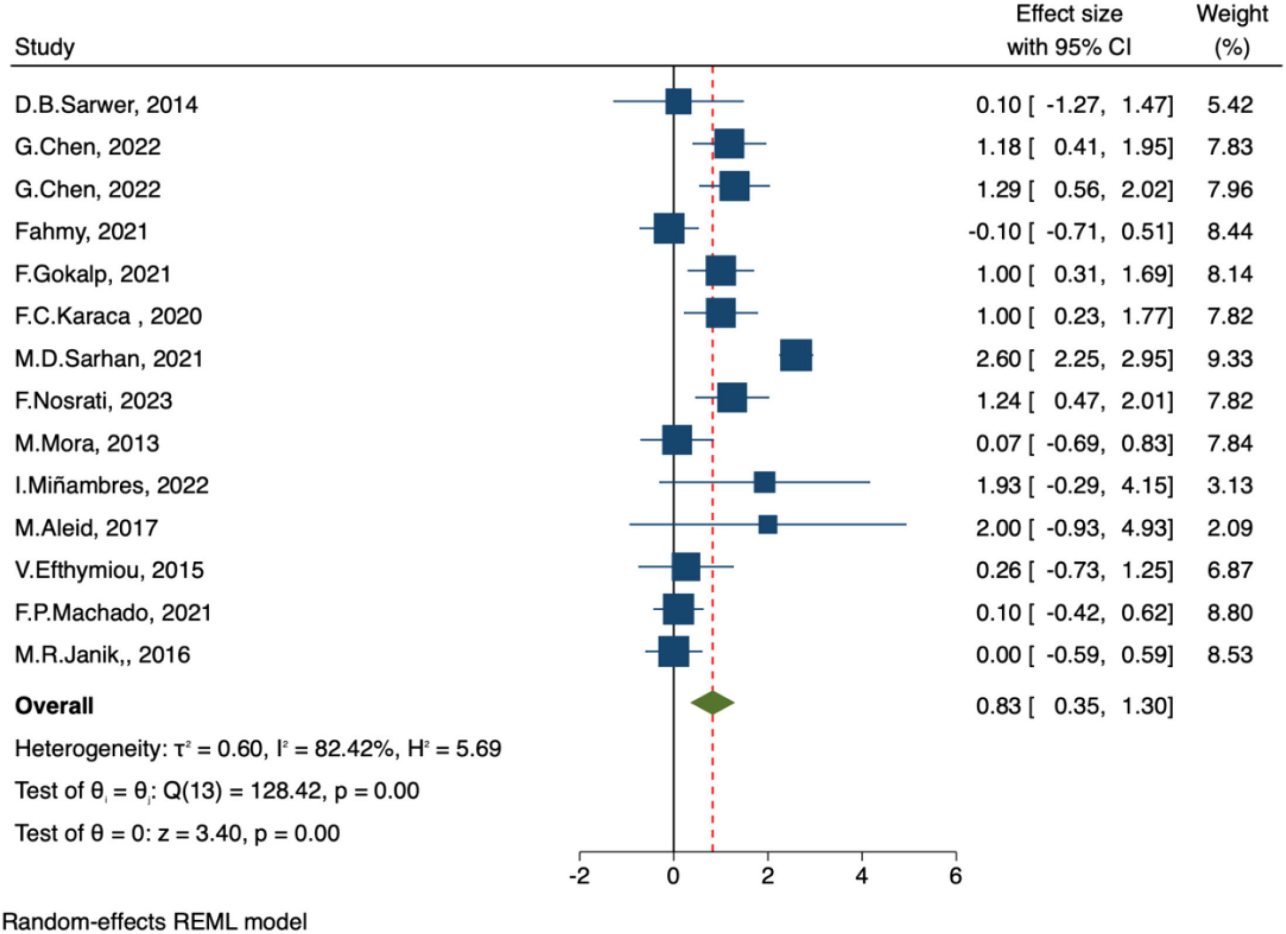
Forest plot-comparison of preoperative and postoperative International Index of Erectile Function-Orgasm Function.

**Figure 6:**
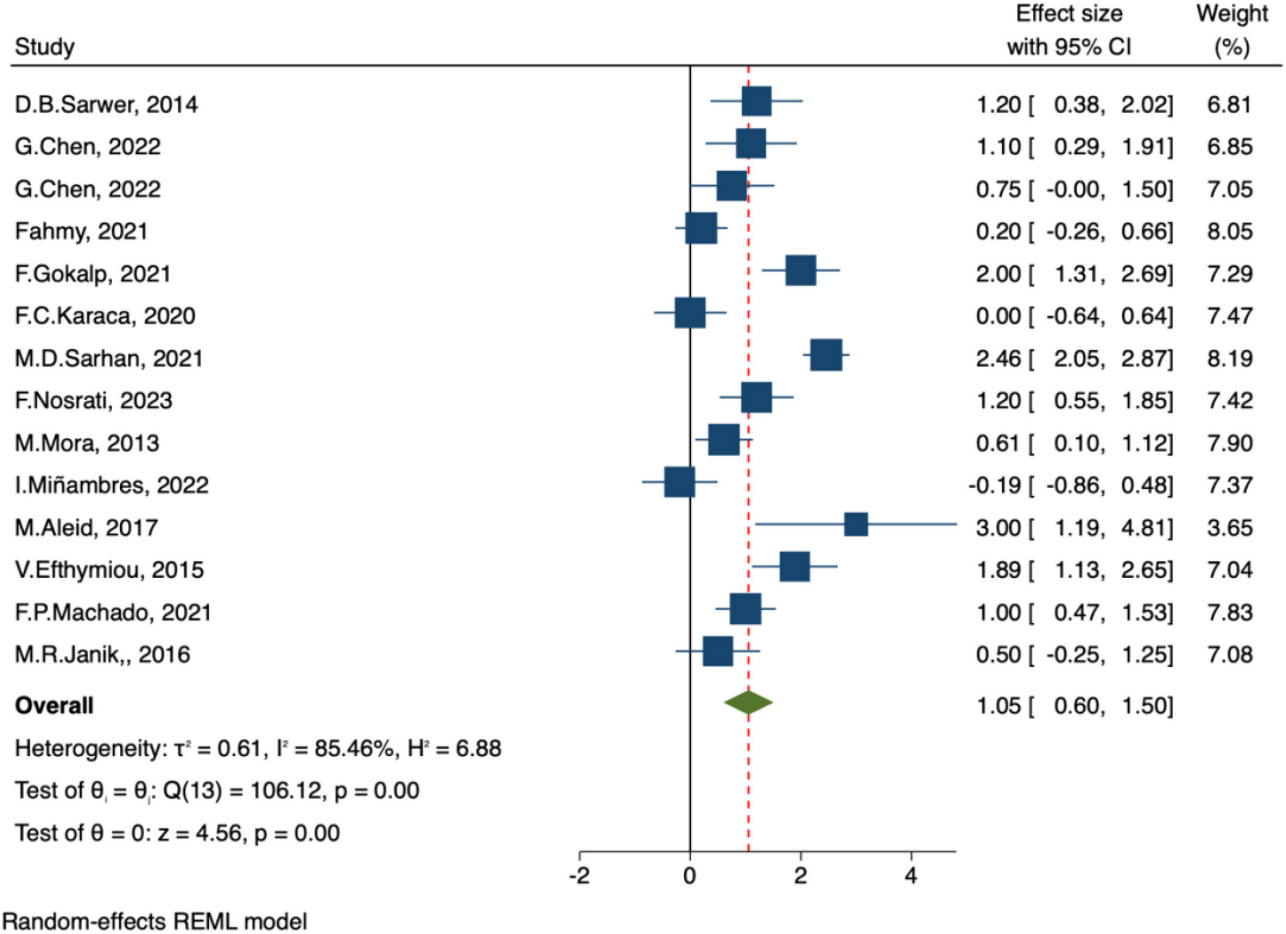
Forest plot-comparison of preoperative and postoperative International Index of Erectile Function-Desire.

A total of 14 studies assessed the impact of bariatric surgery on male orgasm function (IIEF-Orgasm Function) and sexual desire (IIEF-Desire). The pooled results from the random-effects model demonstrated significant improvements after surgery, with IIEF-Orgasm Function: MD = 0.83, 95% CI: 0.35–1.30, p < 0.001 (**Figure 5**), and IIEF-Desire: MD = 1.05, 95% CI: 0.60–1.50, p < 0.001 (**Figure 6**). The heterogeneity was substantial, with I² = 82.4% and I² = 85.46%, respectively, indicating significant between-study variability.

##### IIEF-intercourse satisfaction and IIEF-total satisfaction

**Figure 7:**
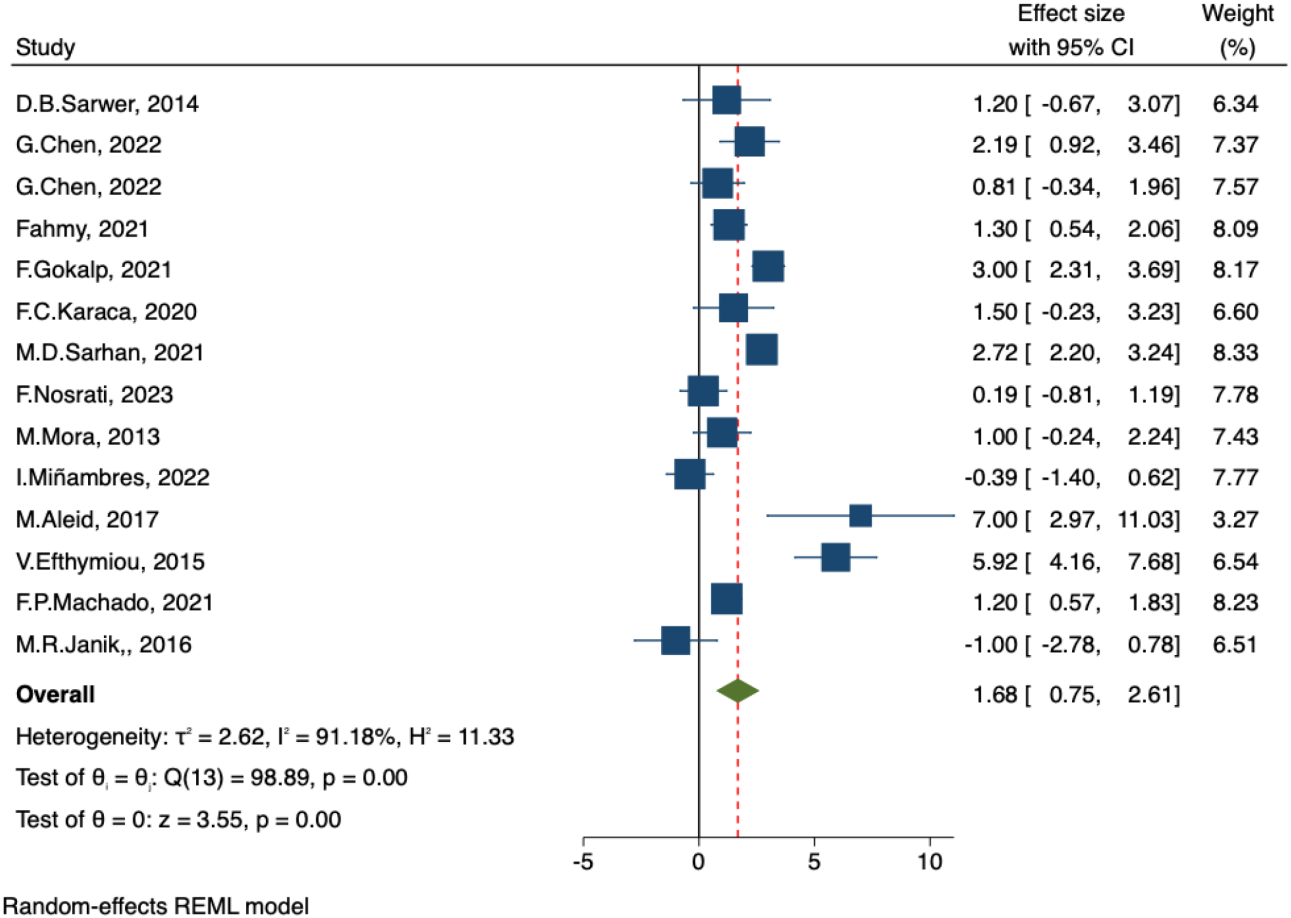
Forest plot-comparison of preoperative and postoperative International Index of Erectile Function-Intercourse Satisfaction.

**Figure 8:**
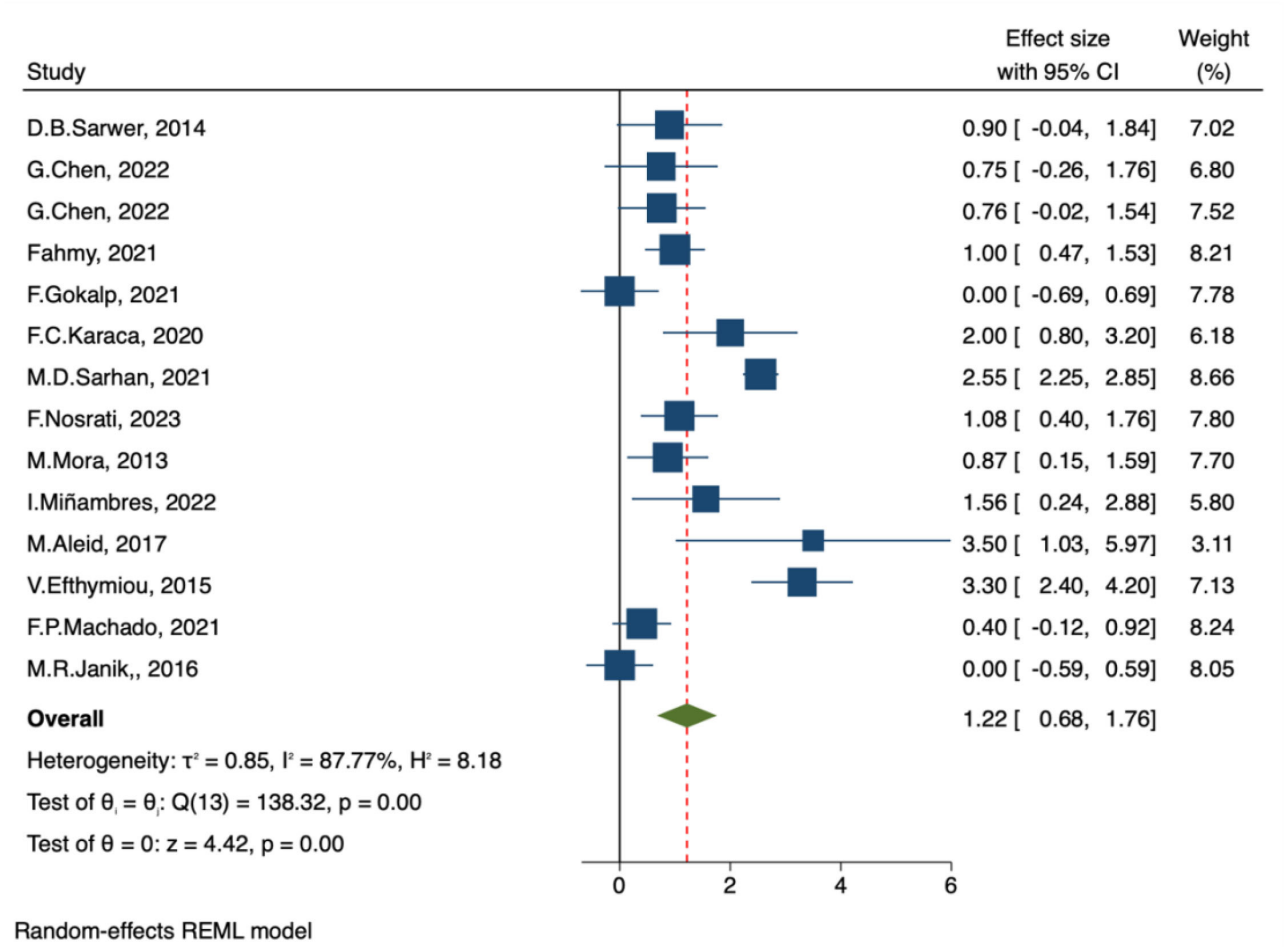
Forest plot-comparison of preoperative and postoperative International Index of Erectile Function-Total Satisfaction.

A total of 14 studies reported changes in intercourse satisfaction and overall satisfaction scores before and after bariatric surgery. The pooled results from the random-effects model indicated significant improvements following surgery, with IIEF-Intercourse Satisfaction: MD = 1.68, 95% CI: 0.75–2.61, p < 0.001 (**Figure 7**) and IIEF-Total Satisfaction: MD = 1.22, 95% CI: 0.68–1.76, p < 0.001 (**Figure 8**). Substantial heterogeneity was observed across studies, with I² = 91.18% and I² = 87.77%, respectively, indicating considerable between-study variability.

To further clarify the differences in improvement across the five domains of the IIEF-15, we conducted a meta-regression analysis. The results demonstrated that improvements were consistent across all domains (erectile function, orgasmic function, sexual desire, intercourse satisfaction, and overall satisfaction). Among them, erectile function showed the greatest improvement (SMD = 0.76), although the differences compared with other domains were not statistically significant (p > 0.05). The ranking of improvements was as follows: erectile function > overall satisfaction > sexual desire > intercourse satisfaction > orgasmic function (**Table 4**).

**Table 4:** Comparison of improvements across IIEF-15 domains after bariatric surgery.

Domain	Pooled SMD (95% CI)	Rank	Difference vs. erectile function	P value
Erectile Function	0.76 (0.47, 1.01)	1	Reference	—
Overall Satisfaction	0.61 (0.26, 0.96)	2	-0.13	0.508
Sexual Desire	0.58 (0.17, 0.99)	3	-0.16	0.443
Intercourse Satisfaction	0.57 (0.17, 0.96)	4	-0.18	0.388
Orgasmic Function	0.44 (0.01, 0.88)	5	-0.31	0.128

#### Secondary outcomes

##### Testosterone and free testosterone

**Figure 9:**
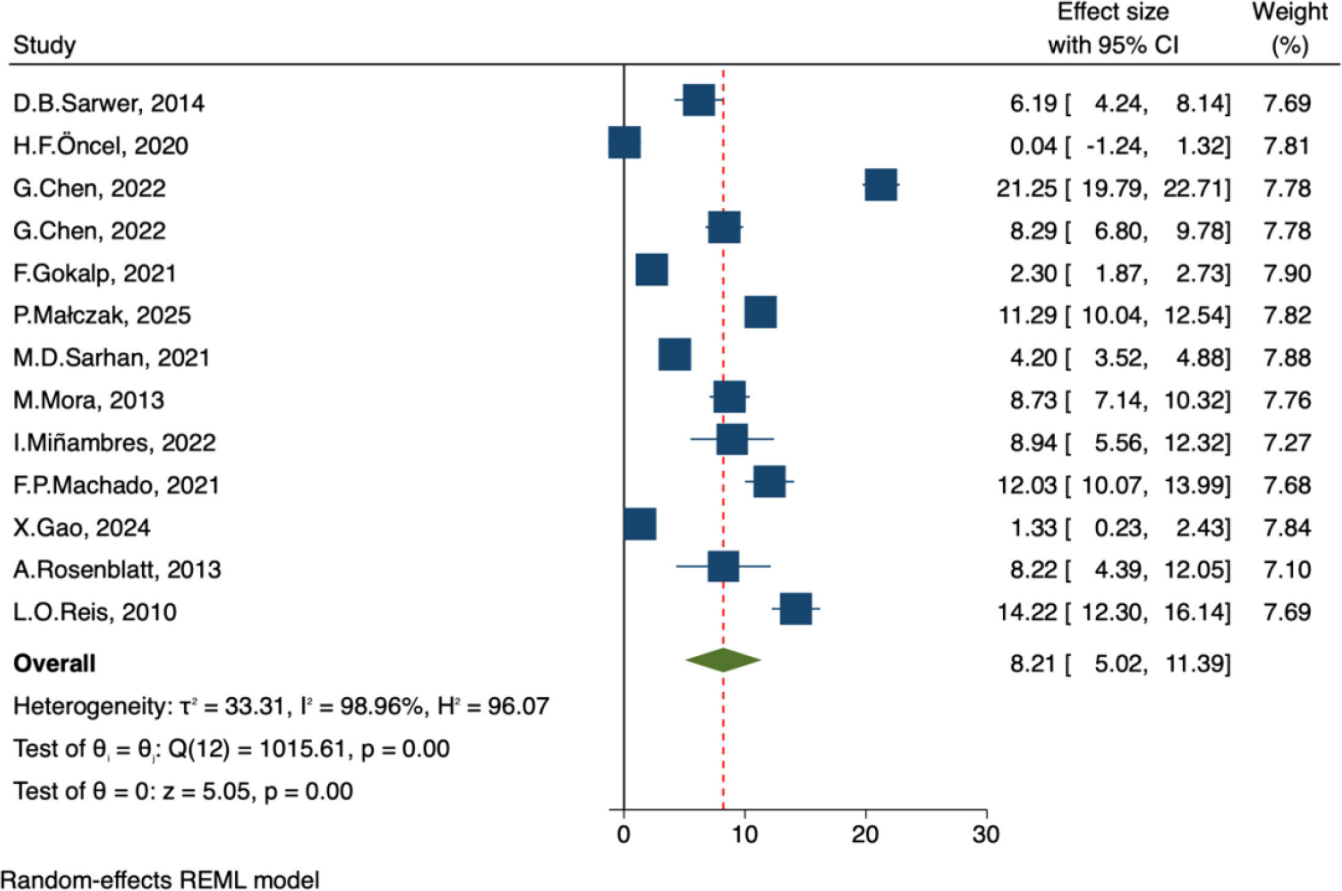
Forest plot-comparison of preoperative and postoperative total testosterone.

**Figure 10:**
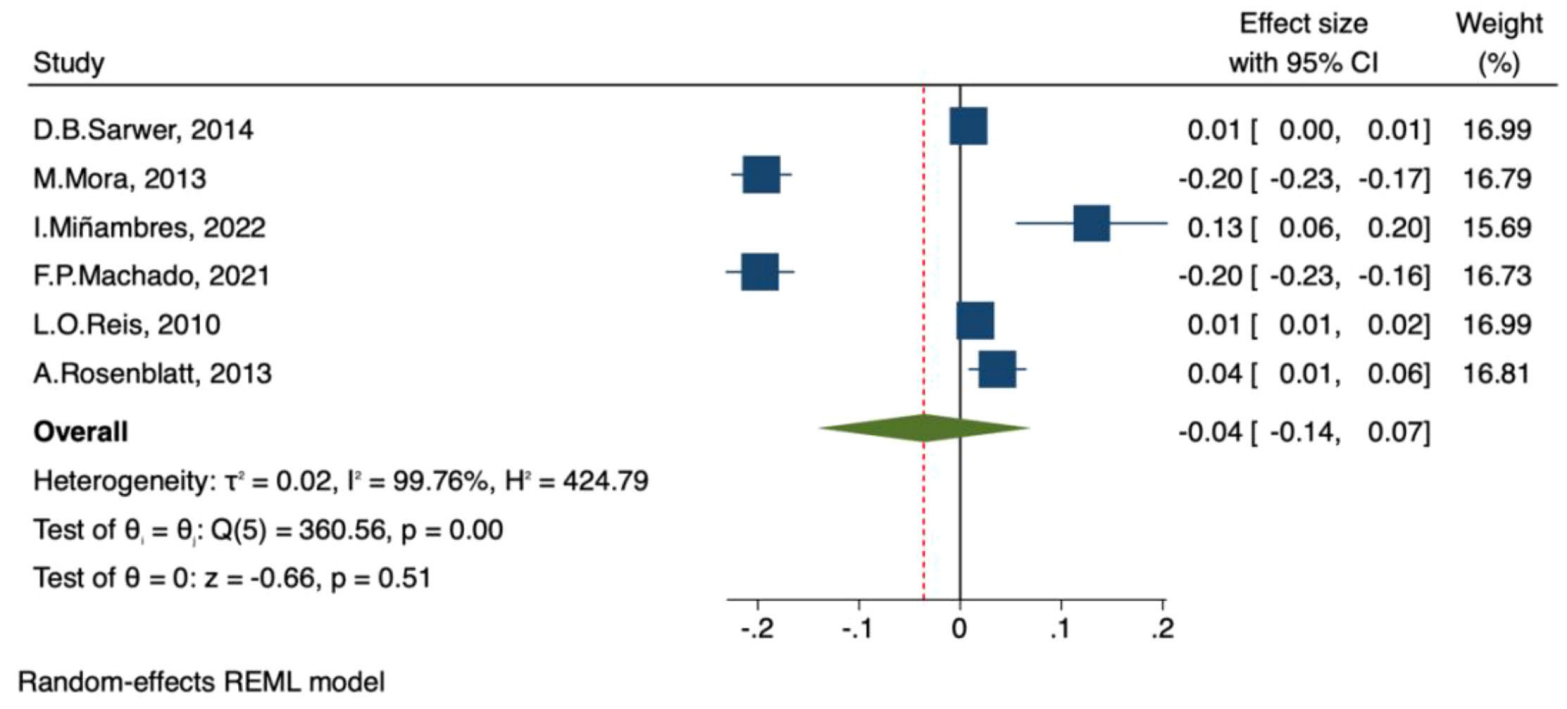
Forest plot-comparison of preoperative and postoperative free testosterone.

A total of 13 studies reported preoperative and postoperative total testosterone (TT) levels, which were reported in different units but standardized to nmol/L. Pooled analysis using a random-effects model showed that bariatric surgery significantly increased TT levels in men (MD = 8.21, 95% CI [5.02, 11.39], p < 0.001). The confidence interval did not cross 0, indicating a statistically significant difference before and after surgery. However, heterogeneity was extremely high (τ² = 33.31, I² = 98.96%, Q(12) = 1015.61, p < 0.001) (**Figure 9**).Six studies reported preoperative and postoperative free testosterone (FT) levels, also standardized to nmol/L. Random-effects model analysis showed no significant change in FT after surgery (MD = -0.04, 95% CI [-0.14, 0.07], p = 0.51). The confidence interval crossed 0, indicating no statistically significant difference. Heterogeneity was also very high (τ² = 0.02, I² = 99.76%, Q(5) = 360.5, p < 0.001) (**Figure 10**).

##### BMI

**Figure 11:**
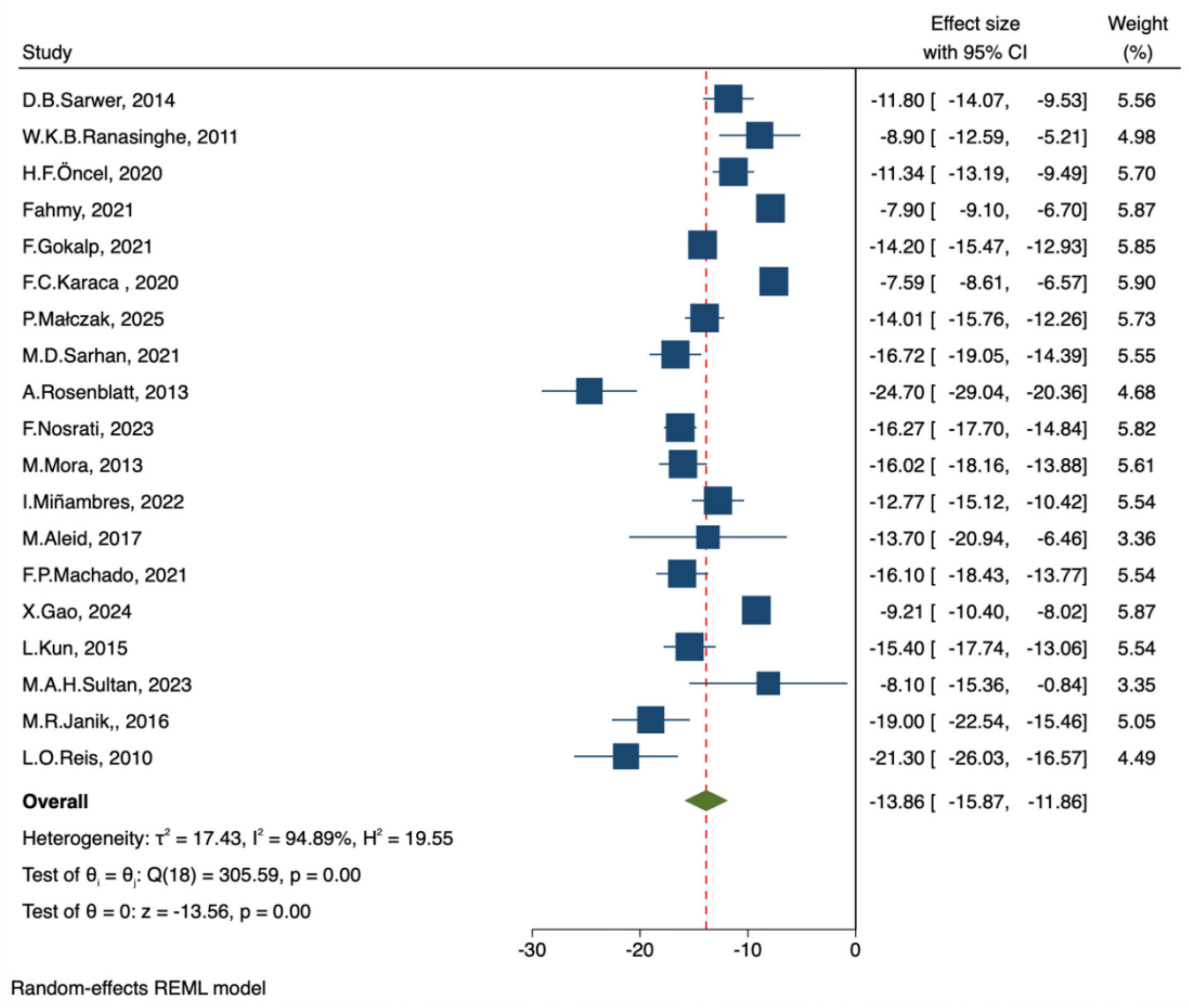
Forest plot-comparison of preoperative and postoperative BMI.

A total of 19 studies reported preoperative and postoperative body mass index (BMI) levels. A meta-analysis using a random-effects model was conducted to evaluate changes in BMI after bariatric surgery. The pooled analysis demonstrated that patients’ BMI was significantly reduced compared with baseline levels (MD = -13.86 kg/m², 95% CI: -15.87 to -11.86, z = -13.56, p < 0.001). This difference was highly statistically significant. Substantial heterogeneity was observed among the included studies (I² = 94.89%, τ² = 17.43, Q = 305.59, p < 0.001) (**Figure 11**).

### Subgroup analysis

For the meta-analysis of IIEF-15, heterogeneity analysis indicated substantial heterogeneity (I² = 87.2%). To further explore potential sources of heterogeneity and to test the robustness of the pooled results, we conducted subgroup analyses according to country, surgical procedure, and study design. All analyses were performed using random-effects models.

**Figure 12:**
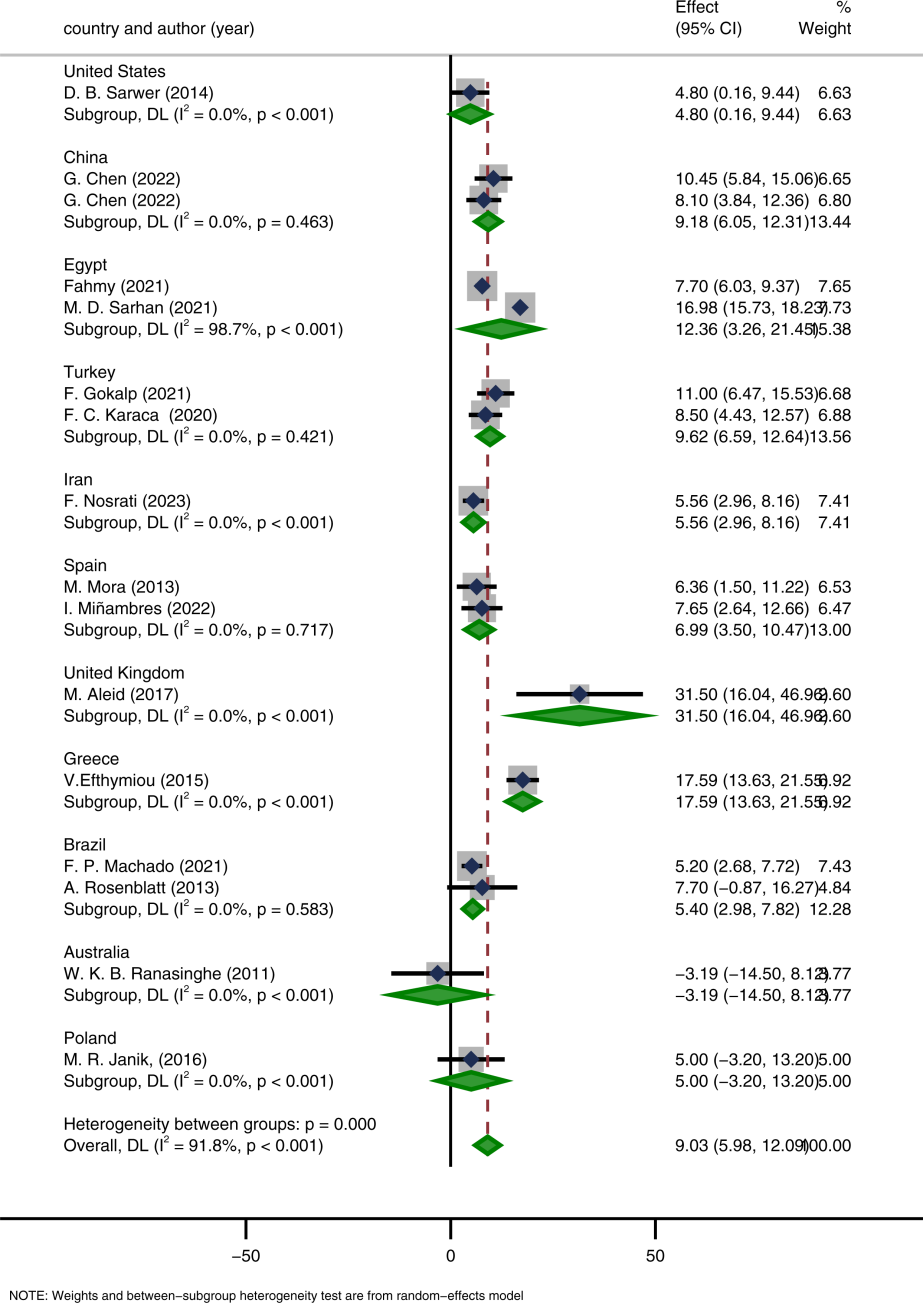
Subgroup analysis based on the country.

When stratified by country, differences in pooled effect sizes were observed across regions. Studies from the United Kingdom reported a significant improvement in IIEF-15 scores after surgery (MD = 31.5, 95% CI: 16.04–46.96, p < 0.001), whereas studies from Australia showed no significant improvement (MD = -3.19, 95% CI: -14.5–8.12). Overall, most countries demonstrated favorable effects of bariatric surgery on male sexual function (**Figure 12**).

**Figure 13:**
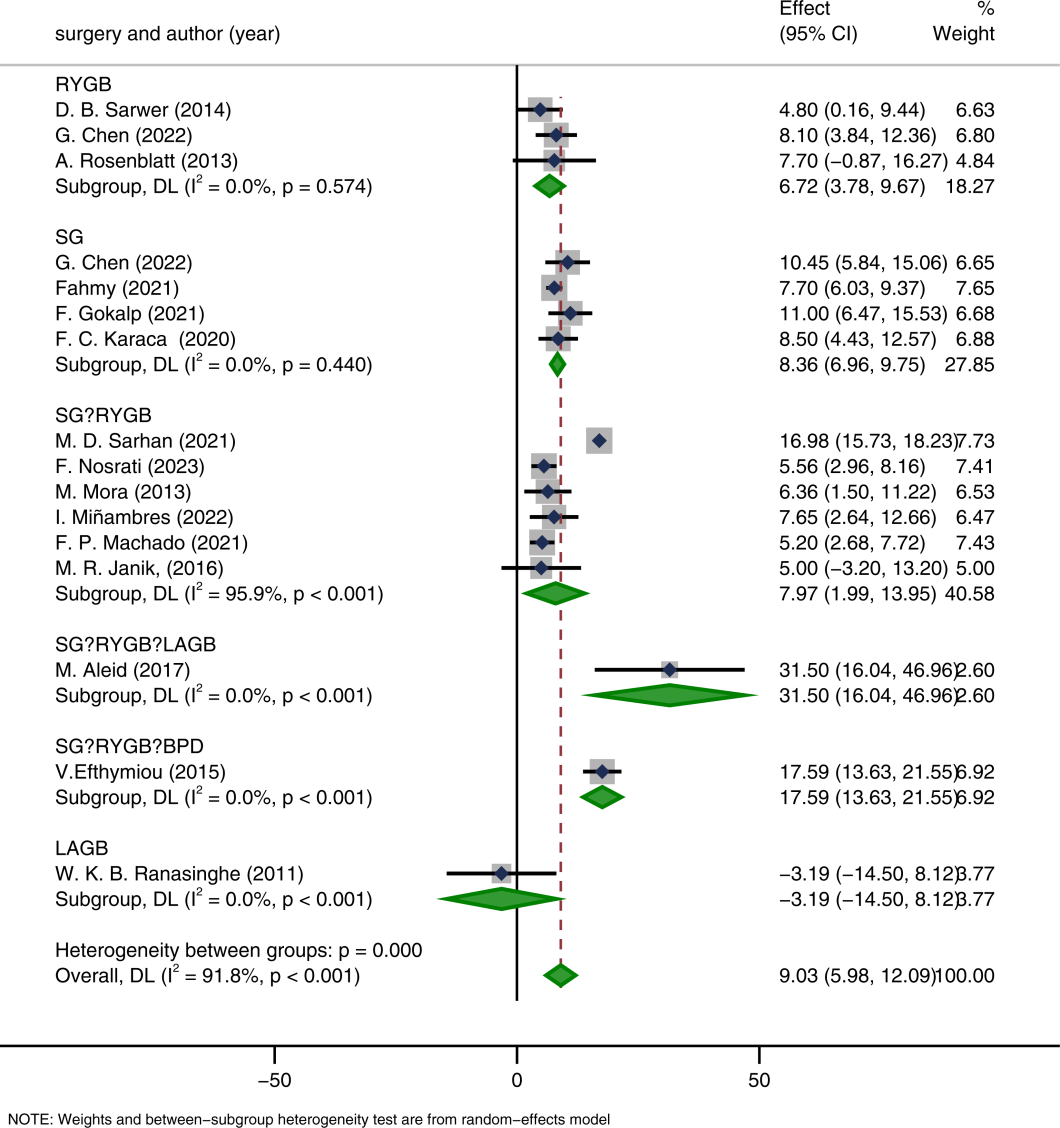
Subgroup analysis based on the surgical procedure.

In the subgroup analysis by surgical procedure, sleeve gastrectomy (SG) was associated with a significant increase in IIEF-15 scores (MD = 8.36, 95% CI: 6.96–9.75, p < 0.001). Roux-en-Y gastric bypass (RYGB) also showed significant improvement (MD = 6.72, 95% CI: 3.78–9.67, p < 0.001). Both the SG and RYGB subgroups showed low heterogeneity (I² = 0.0%, p < 0.001), suggesting good consistency within groups. Notably, studies that included multiple surgical procedures demonstrated comparable magnitudes of improvement, albeit with some differences compared to single-procedure studies (**Figure 13**). Given that some studies reported results for single procedures (SG, RYGB, or LAGB), we further conducted a subgroup difference test using meta-regression, with LAGB as the reference group. The results indicated no statistically significant differences across surgical procedures (p = 0.695) (**Table 5**). As meta-regression is observational at the study level and does not establish causal superiority. Furthermore, the limited number of studies and potential overlap of cohorts may restrict the reliability of comparative inferences.

**Table 5:** Meta-analysis meta-regression results.

Surgery type	Coefficient	Std_err	z	P_value	CI_lower	CI_upper
RYGB	9.989	8.945	1.12	0.264	-7.543	27.521
SG	12.539	8.676	1.45	0.148	-4.467	29.544
SG, RYGB	11.24	8.509	1.32	0.187	-5.437	27.917
SG, RYGB, BPD	20.78	10.149	2.05	0.041	0.887	40.673
SG, RYGB, LAGB	34.69	12.694	2.73	0.006	9.809	59.571
_cons	-3.19	8.13	-0.39	0.695	-19.125	12.746

Test of residual homogeneity: Q_res = chi2(10) = 125.89 Prob > Q_res = 0.0000, Comparison with LAGB.

**Figure 14:**
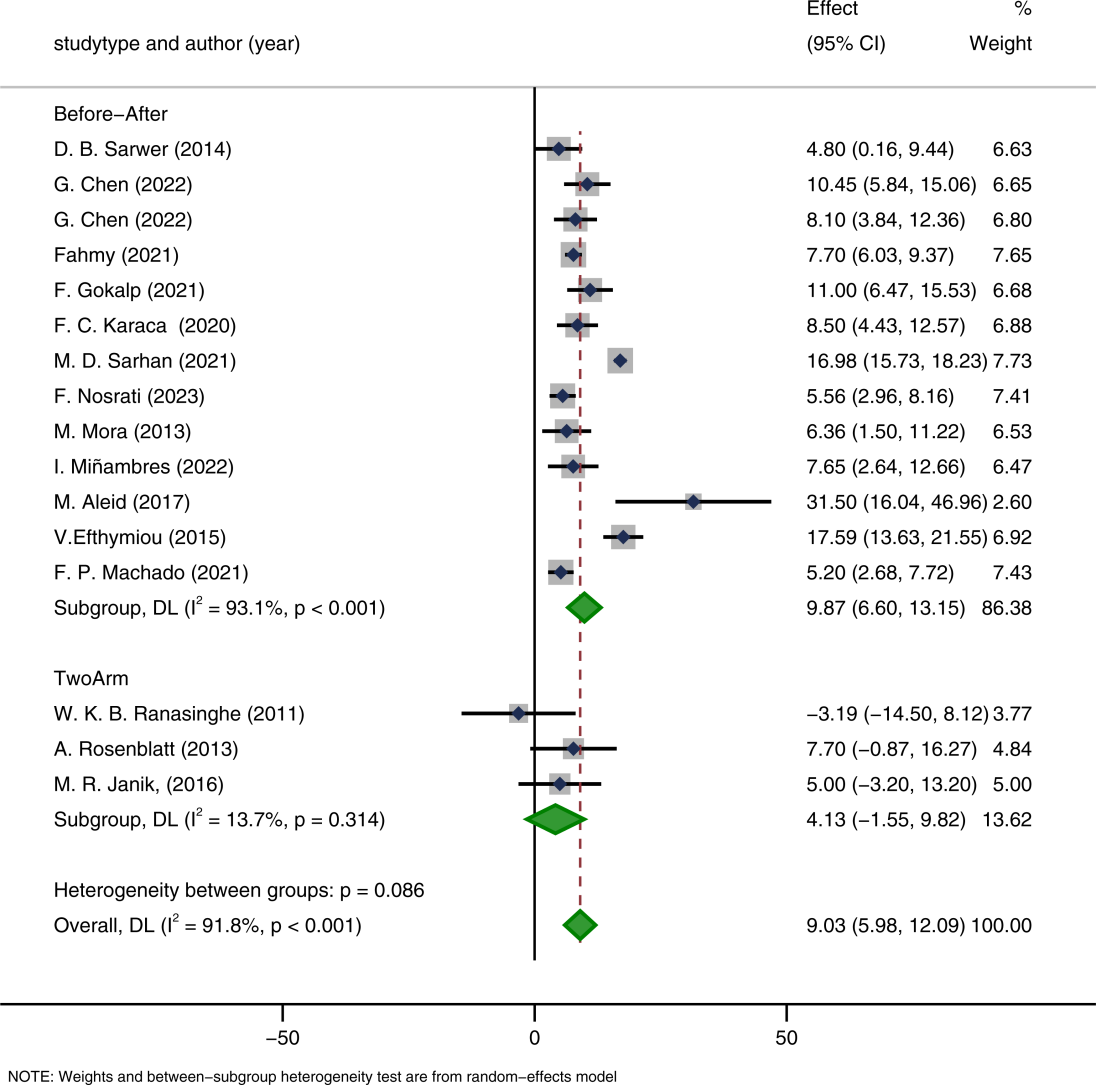
Subgroup analysis based on the study type.

In the subgroup analysis by study design, single-arm before–after studies showed significant improvements in IIEF-15 scores after surgery (MD = 9.87, 95% CI: 6.60–13.15, p < 0.001). Similarly, two-arm comparative studies also indicated an improvement (MD = 4.13, 95% CI: -1.55–9.82, p < 0.001). The heterogeneity in the two-arm studies was 0% (p < 0.001), suggesting high consistency among studies within this subgroup and robust pooled estimates (**Figure 14**).

These findings indicate that regardless of study design, bariatric surgery consistently exerts a positive effect on male sexual function. Overall, the subgroup analyses confirmed the significant improvement of IIEF-15 scores following bariatric surgery, with consistent directions of effect across subgroups, despite some differences in the magnitude of effect among countries, surgical procedures, and study designs. In addition, I² values decreased after some subgroup analyses, suggesting that heterogeneity may partly originate from differences in study populations and surgical procedures.

### Sensitivity analysis and publication bias assessment

To verify the robustness of the meta-analysis results, we performed a leave-one-out sensitivity analysis for the pooled effect size of IIEF-15. The results showed that after sequentially excluding each individual study, the pooled effect size remained stable around MD ≈ 9.51 (95% CI: 6.43–12.59), and the overall trend did not undergo substantial changes (see sensitivity analysis plot). This indicates that no single study had a decisive impact on the overall results, suggesting that the meta-analysis findings based on IIEF-15 are robust and reliable.

**Figure 15:**
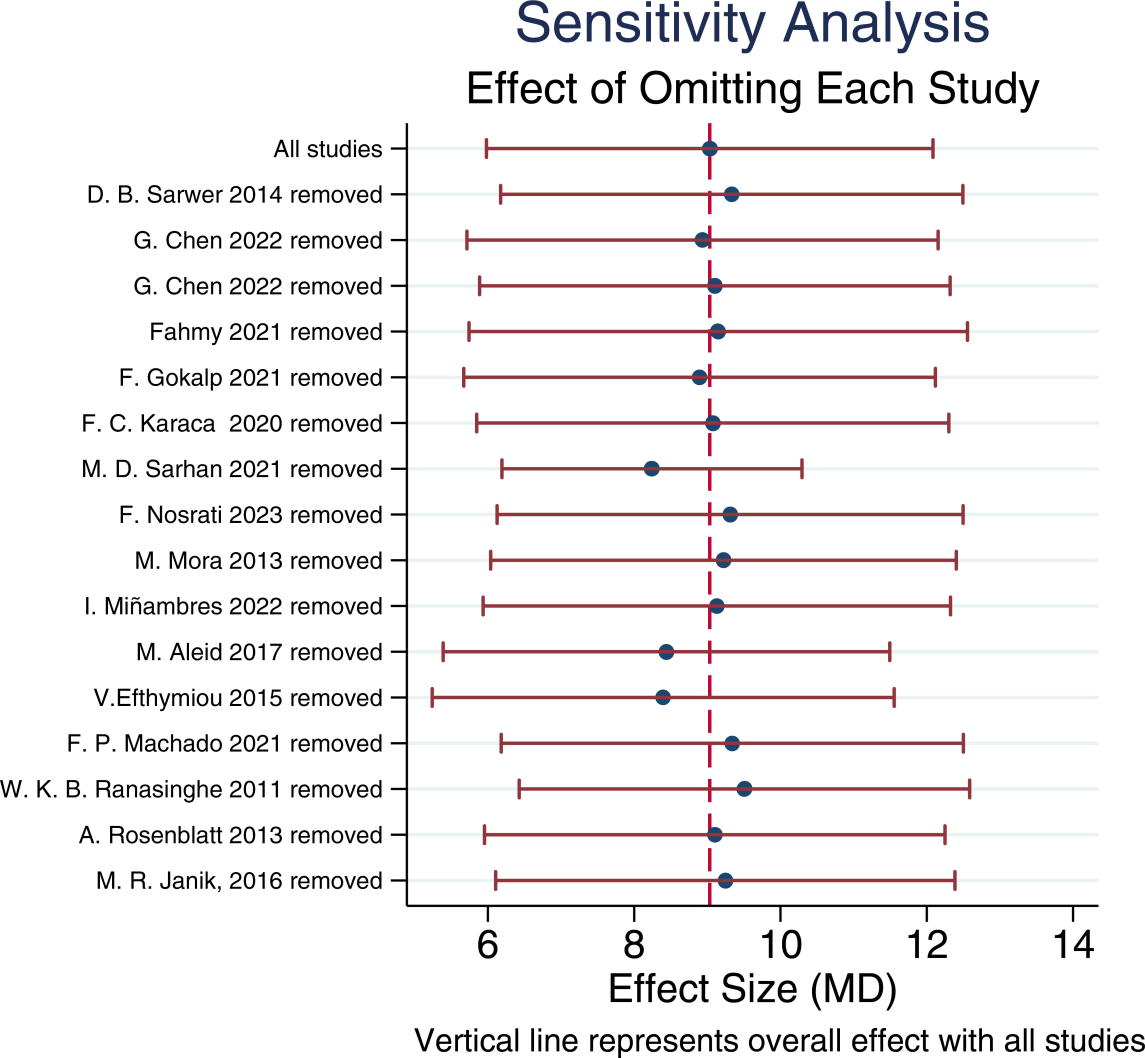
Sensitivity analysis results.

Overall heterogeneity was relatively high (I² ≈ 92%). Sensitivity analysis identified the study by Sarhan et al. (28) as the major contributor to heterogeneity; after its exclusion, I² markedly decreased to 71%, suggesting that this study may be the primary source of heterogeneity (**Figure 15**).

**Figure 16:**
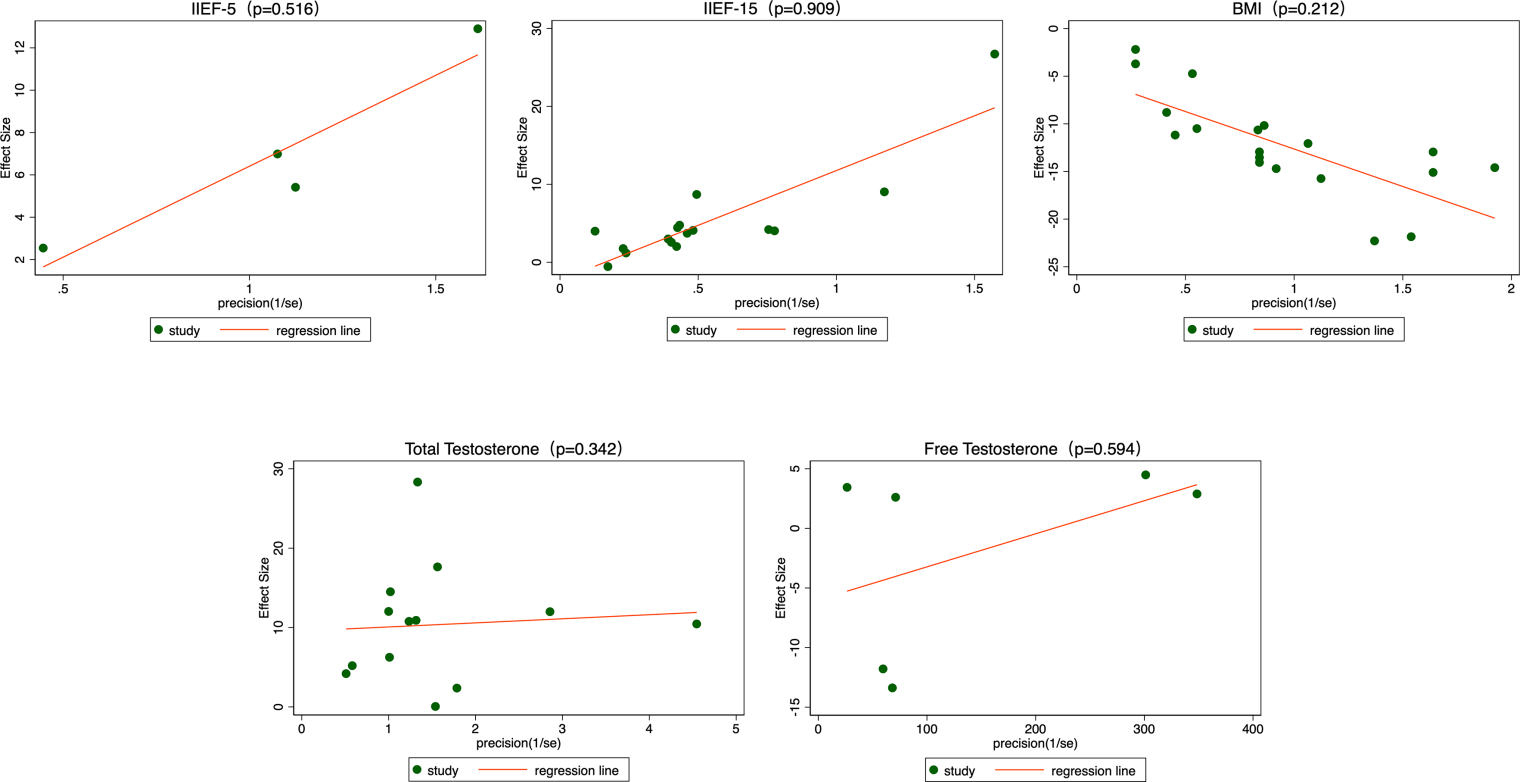
Publication bias - Egger graphs. BMI, body mass index; IIEF, International Index of Erectile Function.

**Figure 17:**
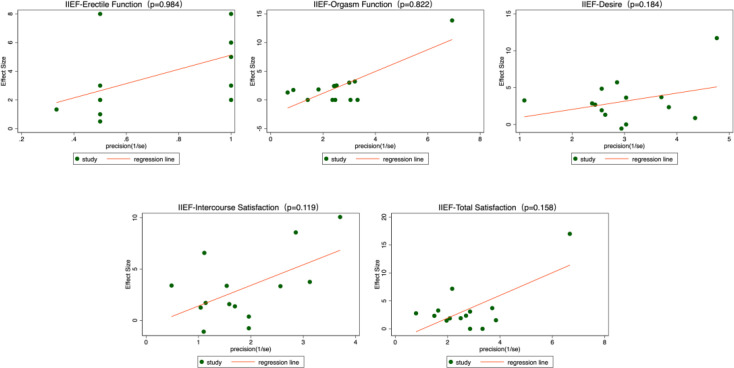
Publication bias - Egger graphs. BMI, body mass index; IIEF, International Index of Erectile Function.

To assess the potential publication bias of this meta-analysis, we performed Egger’s regression test. The results showed that the p-values for all outcomes were greater than 0.05, indicating no significant evidence of publication bias (**Figures 16** and **17**).

## Discussion

With the transformation of the global economy and improvements in living standards, obesity has become an increasingly prominent global health issue (36). Studies have shown that the incidence of erectile dysfunction is significantly higher among overweight and obese patients compared to individuals with normal body weight (37). Currently, bariatric surgery is becoming increasingly widespread and represents the most effective treatment option for many obese patients (38).

The findings of this systematic review and meta-analysis demonstrate that bariatric surgery significantly improves sexual function in obese men. Evidence from four studies using the abridged International Index of Erectile Function (IIEF-5) indicated a significant postoperative increase in overall IIEF-5 scores, suggesting marked improvements in erectile function. Similarly, results from 16 studies using the IIEF-15 also revealed significant enhancement, with positive changes observed across multiple domains, including erectile function, orgasmic function, sexual desire, and intercourse satisfaction. These results are consistent with prior studies, which have likewise reported that bariatric surgery can effectively improve erectile function and overall sexual quality of life in men (39).

In addition, to further clarify the effects of bariatric surgery on specific domains of the IIEF-15, we performed a meta-regression analysis across its five domains. The results indicated that improvements were consistent across erectile function, orgasmic function, sexual desire, intercourse satisfaction, and overall satisfaction. The order of improvement was as follows: erectile function > overall satisfaction > sexual desire > intercourse satisfaction > orgasmic function. This finding suggests that the positive effects of bariatric surgery on male sexual function are multifaceted, with erectile function representing the most pronounced benefit. Nonetheless, due to limited sample size, these inter-domain differences were not statistically significant. Meanwhile, orgasmic function, sexual desire, and satisfaction also demonstrated varying degrees of improvement, though to a relatively smaller extent. These results imply that the recovery of male sexual function depends not only on physiological mechanisms but is also closely associated with psychological improvement, the quality of partner relationships, and other psychosocial factors (40, 41).

Changes in endocrine levels may represent one of the key mechanisms underlying these improvements. Our study found that bariatric surgery significantly increased total testosterone levels in men, a finding consistent with Lee et al. (42), who reported a significant rise in testosterone levels following weight loss. However, free testosterone levels did not change significantly in the present analysis. Possible explanations include alterations in sex hormone-binding globulin (SHBG) (43), Obesity suppresses hepatic SHBG synthesis through hyperinsulinemia and inflammation. Postoperatively, as weight reduction, SHBG levels elevate with an increase in total testosterone. However, free testosterone levels do not increase proportionally due to the increased binding capacity. In addition, heterogeneity in laboratory assays used to measure testosterone across studies may further contribute to variability in FT results. Importantly, improvements in sexual function may occur independently of measurable changes in circulating free testosterone, suggesting that metabolic, vascular, and psychological mechanisms also play substantial roles. Weight reduction relieves the obesity-induced elevation of estrogen, thereby reducing its negative feedback on luteinizing hormone (LH) secretion, ultimately enhancing testosterone production (44–46). Increased testosterone can, in turn, enhance libido and erectile capacity through multiple processes, including stimulating nitric oxide synthase activity and promoting the development, maintenance, and plasticity of cavernous and pelvic ganglion nerves. Androgens are therefore essential for sustaining male sexual desire and erectile function (47). At the same time, we observed a significant reduction in BMI, indicating substantial postoperative weight loss. This marked reduction in body weight contributes to improvements in metabolic syndrome, decreases in insulin resistance, and optimization of vascular endothelial function, thereby indirectly facilitating the recovery of sexual function.

Several obesity-related mechanisms may help explain the observed improvements in sexual function following bariatric surgery. Obesity is commonly associated with pathological changes such as chronic inflammation, endothelial dysfunction, and insulin resistance, all of which contribute to penile vascular injury and erectile dysfunction (48–50). After bariatric surgery, levels of inflammatory cytokines (TNF-α, IL-12, and IL-6) decline, alleviating obesity-induced low-grade metabolic inflammation and improving insulin signaling. This restoration of endothelial nitric oxide (NO) production enhances vasodilation and penile blood flow, thereby facilitating the recovery of erectile function (51, 52).In addition, weight loss helps rebalance the estrogen-androgen axis, leading to increased testosterone levels. From a psychological perspective, obesity often negatively impacts body image and self-esteem, with depressive symptoms being more prevalent in this population—factors that can suppress sexual desire and sexual activity (53). Following weight reduction, improvements in self-confidence, body image, and psychological well-being (54) further contribute to the restoration of sexual function. Taken together, bariatric surgery promotes comprehensive improvements in sexual function among obese men through multiple synergistic pathways, including optimizing the endocrine environment, reducing chronic inflammation, and enhancing psychological health.

Subgroup analyses were conducted to explore potential sources of heterogeneity and to verify the robustness of the findings. Analyses were stratified by country, surgical procedure, and study design. The results indicated regional variations in effect estimates; for example, studies from the United Kingdom reported significant improvements in IIEF-15 scores (24), whereas those from Australia showed no significant changes (18). All surgical procedures demonstrated positive effects: both the sleeve gastrectomy (SG) and Roux-en-Y gastric bypass (RYGB) groups exhibited significant increases in IIEF-15 scores, with low within-group heterogeneity, suggesting consistent benefits of these two procedures for improving sexual function. Meta-regression analysis showed that both procedures resulted in greater improvement in sexual function compared with AGB, but the differences among surgical procedures were not statistically significant (p = 0.695), indicating that the effects of different bariatric procedures on sexual function improvement were generally comparable, consistent with the findings of Chen et al. (30). Theoretically, RYGB may confer slightly greater metabolic benefits by modulating gut hormones and enhancing insulin sensitivity (55), whereas sleeve gastrectomy (SG) promotes rapid weight loss and improves inflammation and endothelial function primarily through restriction of food intake and alterations in the gut microbiota (56). In contrast, adjustable gastric banding (AGB) generally yields a smaller degree of weight reduction and exerts limited effects on hormonal regulation, which may account for its relatively modest impact on sexual function improvement. With respect to study design, both single-arm before–after studies and two-arm comparative studies demonstrated significant postoperative improvements in sexual function. Notably, heterogeneity within the two-arm subgroup was 0%, suggesting that study design may be one of the contributors to the overall heterogeneity. Overall, the results of subgroup analyses were directionally consistent, all pointing toward the beneficial effects of bariatric surgery on male sexual function. Although effect sizes varied across subgroups—likely related to baseline characteristics, choice of surgical technique, and follow-up duration—these differences did not alter the robustness of the overall conclusion.

Substantial statistical heterogeneity was observed across several pooled analyses and warrants cautious interpretation. This heterogeneity may be attributable to differences in baseline severity of sexual dysfunction, variability in follow-up duration, and the inclusion of mixed bariatric procedures within certain cohorts. Patients with more severe baseline dysfunction may experience greater absolute improvements, thereby contributing to between-study variability. Differences in the timing of outcome assessment may further influence the magnitude of reported effects.

This study has several limitations. An important limitation of the present meta-analysis lies in the inherent weaknesses of the included study designs. The majority of studies were observational or single-arm before–after investigations, lacking concurrent control groups. Such designs are susceptible to regression to the mean, placebo effects, and unmeasured temporal confounding, which may result in overestimation of treatment effects. Therefore, although consistent improvements were observed, causal inference remains limited, and well-designed randomized controlled trials are required to provide more definitive evidence.

Beyond these considerations, several methodological limitations should also be acknowledged. Long-term follow-up data remain scarce, and the durability of sexual function improvement beyond the early postoperative period is uncertain. Most studies focused primarily on patient-reported sexual outcomes, with limited integration of psychosocial variables, relationship dynamics, or partner-reported outcomes, despite the inherently relational nature of sexual function. Furthermore, few studies performed stratified analyses according to key baseline characteristics such as age, severity of erectile dysfunction, or metabolic comorbidities (e.g., diabetes), limiting the ability to identify subgroups deriving the greatest benefit.

Finally, variability in laboratory assessment methods represents another important concern. Differences in testosterone assay techniques and inconsistent reporting of sex hormone-binding globulin (SHBG) complicate the interpretation of hormonal changes. Because weight loss and improved insulin sensitivity may increase SHBG concentrations, total testosterone levels can rise without proportional changes in biologically active free testosterone. In the absence of standardized measurement protocols, including SHBG-adjusted or calculated free testosterone assessments, comparisons across studies should be interpreted cautiously, and clearer mechanistic insights may remain limited.

Future well-designed randomized controlled trials with extended follow-up are needed to strengthen causal inference and to determine the durability of sexual function improvements over time. In addition, stratified analyses based on age, baseline erectile dysfunction severity, and metabolic comorbidities may help identify patient subgroups that derive the greatest benefit from bariatric surgery. Taken together, such efforts will further refine patient selection, clarify underlying mechanisms, and enhance the clinical translation of bariatric surgery in the management of male sexual dysfunction.

## Conclusion

This systematic review and meta-analysis demonstrate that bariatric surgery significantly improves sexual function in obese men. Regardless of whether the IIEF-5 or IIEF-15 was used as the assessment tool, surgery was associated with a marked postoperative increase in sexual function scores. All five domains of the IIEF-15 (erectile function, orgasmic function, sexual desire, intercourse satisfaction, and overall satisfaction) showed consistent improvement, with erectile function demonstrating the greatest effect, although differences among domains were not statistically significant. In terms of hormones, bariatric surgery significantly increased total testosterone levels, while free testosterone remained unaffected. BMI was substantially reduced, underscoring the important role of surgery in improving metabolic status. Subgroup analyses revealed consistent positive effects of bariatric surgery on male sexual function across different countries, surgical procedures, and study designs. Sensitivity analyses and publication bias assessments indicated that the findings are robust and reliable. Overall, bariatric surgery not only facilitates weight control but may also enhance quality of life in obese men by improving sexual function and endocrine profiles.

## Data Availability

The original contributions presented in the study are included in the article/supplementary material. Further inquiries can be directed to the corresponding authors.
